# Functional and Anatomical Characterization of Corticotropin-Releasing Factor Receptor Subtypes of the Rat Spinal Cord Involved in Somatic Pain Relief

**DOI:** 10.1007/s12035-021-02481-z

**Published:** 2021-07-31

**Authors:** Shaaban A. Mousa, Mohammed Shaqura, Baled I. Khalefa, Li Li, Mohammed Al-Madol, Sascha Treskatsch, Michael Schäfer

**Affiliations:** 1 Department of Anesthesiology and Operative Intensive Care Medicine, Freie Universität Berlin, Humboldt-Universität zu Berlin, and Berlin Institute of Health, Charité – Universitätsmedizin Berlin, Charité Campus Benjamin Franklin, Hindenburgdamm 30, 12203 Berlin, Germany; 2 Zoology Department, Science Faculty, AL-Zintan University, Alzintan, Libya

**Keywords:** Somatic pain, Corticotropin-releasing factor, Spinal cord, ENK-immunoreactive interneurons, Immunofluorescence

## Abstract

Corticotropin-releasing factor (CRF) orchestrates our body’s response to stressful stimuli. Pain is often stressful and counterbalanced by activation of CRF receptors along the nociceptive pathway, although the involvement of the CRF receptor subtypes 1 and/or 2 (CRF-R1 and CRF-R2, respectively) in CRF-induced analgesia remains controversial. Thus, the aim of the present study was to examine CRF-R1 and CRF-R2 expression within the spinal cord of rats with Freund’s complete adjuvant-induced unilateral inflammation of the hind paw using reverse transcriptase polymerase chain reaction, Western blot, radioligand binding, and immunofluorescence confocal analysis. Moreover, the antinociceptive effects of intrathecal (i.t.) CRF were measured by paw pressure algesiometer and their possible antagonism by selective antagonists for CRF-R1 and/or CRF-R2 as well as for opioid receptors. Our results demonstrated a preference for the expression of CRF-R2 over CRF-R1 mRNA, protein, binding sites and immunoreactivity in the dorsal horn of the rat spinal cord. Consistently, CRF as well as CRF-R2 agonists elicited potent dose-dependent antinociceptive effects which were antagonized by the i.t. CRF-R2 selective antagonist K41498, but not by the CRF-R1 selective antagonist NBI35965. In addition, i.t. applied opioid antagonist naloxone dose-dependently abolished the i.t. CRF- as well as CRF-R2 agonist-elicited inhibition of somatic pain. Importantly, double immunofluorescence confocal microscopy of the spinal dorsal horn showed CRF-R2 on enkephalin (ENK)-containing inhibitory interneurons in close opposition of incoming mu-opioid receptor-immunoreactive nociceptive neurons. CRF-R2 was, however, not seen on pre- or on postsynaptic sensory neurons of the spinal cord. Taken together, these findings suggest that i.t. CRF or CRF-R2 agonists inhibit somatic inflammatory pain predominantly through CRF-R2 receptors located on spinal enkephalinergic inhibitory interneurons which finally results in endogenous opioid-mediated pain inhibition.

**Supplementary Information:**

The online version contains supplementary material available at 10.1007/s12035-021-02481-z.

## Introduction

Corticotropin-releasing factor (CRF) is a 41-amino-acid peptide which modulates endocrine, autonomic, and behavioral activity to orchestrate the body’s response to acute and chronic stressful stimuli in order to maintain homeostasis [1]. Pain is such a stressful stimulus that triggers intrinsic compensatory mechanisms through activation of CRF receptors [2–4]. Since many of the physiological responses to stress can be reproduced by the administration of exogenous CRF, Hargreaves et al. [5] investigated for the first time in rats the potential antinociceptive effects of intravenously injected CRF. This showed a fivefold increase in endogenous ß-endorphin plasma levels and a significantly increased threshold to a 50 °C hot plate stimulus. Consistently, a randomized, double-blind placebo-controlled crossover study investigated the pain relief through i.v. injection of 100 µg CRF in 14 patients undergoing third molar extraction. Injecting i.v. CRF 60 min after dental surgery led to a significantly reduced pain intensity and a twofold increase in ß-endorphin plasma levels compared to placebo [5]. In contrast, testing the potential antinociceptive effects of i.v. 100 µg CRF to heat stimuli in 18 healthy volunteers [6, 7] in a double-blind, cross-over and placebo-controlled study design failed to show any effect, most likely due to the lack of persistent ongoing pain.

In addition to somatic pain, CRF seems to intervene in the regulation of visceral pain [8]. Indeed, intracerebroventricular administration of CRF resulted in a significant inhibition of the nociceptive visceromotor response to colorectal distension [9]. Moreover, systemic application of a CRF-R2 agonist inhibited an increase in the spinal activity marker ERK1/2 and prevented a nociceptive visceromotor response to colorectal distension [10]. However, previous studies suggest that CRF-R1 and CRF-R2 activation may have opposing roles in the regulation of visceral pain [8] and the net result of CRF receptor activation may depend on whether the receptors are localized in the brain [11], spinal cord [10], peripheral tissues [8] or immune cells [12]. A growing body of evidence suggests a close link between the pathological condition of the irritable bowel syndrome and the CRF system [13]. Moreover, the accumulating data from the clinical trials confirmed the regulatory role of CRF in pain control of patients with visceral pain [14], postoperative joint pain [15], and fibromyalgia [16, 17].

Our previous studies showed the involvement of spinal CRF receptors in somatic pain modulation through endogenous opioid peptides [18]. However, the biological activities of CRF in various pathophysiological conditions are circumstantial and remain controversial [19]. This diverse effect may be due to the different types of CRF receptors. Indeed, the effects of CRF are mediated by two distinct membrane receptors, the CRF-R1 and CRF-R2, which differ in their anatomical distribution and pharmacological characteristics [20, 21]. Therefore, the main goal of the present study was to determine the CRF-R1 and CRF-R2 distribution in the rat spinal cord and to investigate their contribution to the modulation of inflammatory pain.

## Materials and methods

### Animals

Experiments were conducted in male Wistar rats (200–250 g) (Charité-Universitätsmedizin Berlin, Campus Benjamin Franklin, Berlin, Germany). Rats were housed individually in cages and maintained on a 12 h light/dark schedule with food pellets and water ad libitum. Room temperature was maintained at 22 ± 0.5 °C and at a relative humidity between 60 and 65%. Experiments and animal care were performed according to the European Directive (2010/63/EU) introducing new animal welfare and care guidelines and were approved by the local animal care committee of the Senate of Berlin, Germany (Landesamt für Arbeitsschutz, Gesundheitsschutz und Technische Sicherheit, Berlin) (Animal protocol G0045/14). All efforts were made to minimize the number of animals used and their suffering.

### Induction of inflammation

Under brief anesthesia with isoflurane (Willy Rüsch GmbH, Böblingen, Germany), rats received an intraplantar (i.pl.) injection of 0.15 ml FCA into the right hind paw. This treatment consistently produces a localized inflammation of the inoculated paw as reflected by an increase in paw volume, paw temperature and infiltration with various types of immune cells as previously described [22]. The conventional PCR, radioligand binding assay, algesiometric testing, and western blot, were performed 4 days after induction of FCA inflammation, in line with previous investigations [18, 20, 22, 23].

### Surgery to implant i.t. catheter

The intrathecal catheterization (i.t.) was performed as previously described [24, 25]. Briefly, an incision was made at the L3–L4 level. The catheter was inserted through a needle at the L4–L5 vertebra. Keeping the angle of the needle parallel with the dorsal surface, the catheter was carefully pushed upward to reach L4 at the lumbar enlargement. The needle was carefully removed and the catheter was sealed with glue to the tissue to secure it. Then, saline was injected intrathecally in a volume of 10 µl to flush the catheter. Another skin incision was made at the neck of the animal and the catheter was tunneled under the skin and pulled out at the neck, after which incisions were sutured. Animals showing signs of neurological damage were immediately excluded from the study. The intrathecal location of the catheter was confirmed by administration of 10 µl of lidocaine 2% flushed with 10 µl of saline. Lidocaine but not saline caused reversible bilateral hindlimb paresis. The animals were allowed 2 days to recover. Drugs were injected intrathecally in a volume of 10 µl followed by 10 µl of vehicle to flush the catheter. All rats were investigated for correct catheter position in relation to the spinal cord on post-mortem laminectomy.

### Drugs

The following drugs were used: rat/human CRF (Sigma-Aldrich, St. Louis, MO, USA); CRF-R2 agonist urocortin-2 (Ucn-2), CRF-R2 antagonist K41498; CRF-R1 antagonist NBI35965 (Bio-Techne GmbH, Wiesbaden-Nordenstadt, Germany). Doses were calculated as the free base and drugs were dissolved in isotonic saline as vehicle. For each dose a separate group of animals (n = 6) was used. Drugs were administered during brief isoflurane anesthesia.

### CRF-R1 and CRF-R2 mRNA detection by conventional PCR

Conventional PCR analysis for CRF-R1 and CRF-R2 specific mRNA from rat dorsal root ganglia was performed as described previously [26]. Total RNA was extracted from L3-5 dorsal root ganglia of Wistar rats (n = 5 per experimental group) using RNeasy Kit (Qiagen, Hilden, Germany). 0,5 µl (25 pmol) oligo(dT) and 2 µl (200 pmol) random primers were added up to 1 μg total RNA, incubated at 37 °C for 15 min, then at 85 °C for 5 s, finally at 4 °C for transfer onto ice (according to TaKaRa® manual). cDNA was stored at − 20 °C. The following specific primers were used: for CRF-R1, forward primer: ACACTACCATGTTGCAGTC, reverse primer: GAACATCCAGAAG AAGTTGG (Ensembl, Accession Nr: NM_030999); for CRF-R2, forward primer: CACACTGTGAACCCATTT TGG, reverse primer: GATGAGTTGCAGCAGG (Ensembl, Accession NM_022714). Conventional PCR was performed with a Maxima Hotstart Green Enzyme Kit (Thermo Fisher Scientific GmbH Berlin, Germany). Amplification was carried on an Eppendorf PCR-Cycler Vapo Protect **(**Eppendorf Vertrieb Deutschland GmbH, Wesseling-Berzdorf) out for 40 cycles**,** each consisting of 30 s at 95 °C and of 30 s at 60 °C and 30 s at 72 °C. Specific bands were visualized on 2% agarose gel plus 0.01% ethidium bromide; the entire PCR product of 20 μl migrated for 40 min at 100 V in the BioRad chamber system with 1 × TAE buffer. The imaging was performed on a Gel Doc EZ Imager (Bio-Rad Laboratories GmbH, Feldkirchen, Germany).

### Radioligand binding assay

The following experiments were aimed to identify CRF-R1 or CRF-R2 specific binding sites in membrane preparations of rat spinal cord similar to our previous binding studies [27, 28]. Membrane preparations from Wistar rats were prepared by homogenizing them in cold assay buffer (50 mM Tris–HCl, 1 mM EGTA, 5 mM MgCl_2_, pH 7.4) and were centrifuged at 48,000 × *g* at 4 °C for 20 min. The pellet was resuspended in assay buffer followed by 10 min incubation at 37 °C to remove endogenous ligands. The homogenates were centrifuged again and resuspended in assay buffer. Membranes were aliquoted and stored at − 80 °C [28].

Displacement binding experiments were performed using [125]-iodinated CRF (Specific Activity 1.0 nmol, PerkinElmer, Germany). 150 µg of membrane protein was incubated with [125]-iodinated CRF (1.0 µmol) displaced with 10^−13^–10^−5^ M of CRF-R2 antagonist K41498 or CRF-R1 antagonist NBI35965 for 1 h at 22 °C in a total volume of 1 ml of binding buffer (50 mM Tris–HCl, 5 mM EDTA, 5 mM MgCl2, 100 mM NaCl, 0.2% bovine serum albumin). Nonspecific binding was defined as radioactivity remaining bound in the presence of 10 µM unlabelled CRF. At the end of the incubation period, bound and free ligands were separated by rapid filtration over GF/C filters under vacuum using a Brandel cell harvester (Gaithersburg, MD, USA). Filters were washed three times with 4 ml of cold buffer (50 mM Tris–HCl, pH 7.4). Bound radioactivity was determined by liquid scintillation spectrophotometry after overnight extraction of the filters in 3 ml of scintillation fluid [28]. All experiments were performed in duplicate and carried out at least four times. Nonspecific binding was subtracted from all [125]-iodinated CRF data. IC50 values in saturation binding assays were determined by nonlinear regression analysis of concentration-effect curves using GraphPad Prism (GraphPad Software Inc., CA, USA).

### Algesiometric testing

Nociceptive thresholds were assessed by paw pressure algesiometer test (modified Randall-Selitto test). Animals (n = 6 per group) were gently restrained under paper wadding and incremental pressure was applied via a wedge-shaped, blunt piston onto the dorsal surface of the hind paw by means of an automated gauge (Ugo Basile). The pressure required to elicit paw withdrawal, the paw pressure threshold (PPT), was determined. A cutoff of 250 g was used. Three consecutive trials, separated by intervals of 10 s, were conducted and the average was determined. Baseline PPT were tested before and 4 days after inoculation with FCA. The same procedure was performed on the contralateral side; the sequence of sides was alternated between subjects to preclude order effects. In all behavioral experiments, drugs were prepared by a different person (M. Sh.) and the examiners (B.N. and L.L.) were unaware of the treatment that each animal received by chance.

### Western blot

Spinal cord from adult rats were solubilized and extracted for immunoblotting investigations as previously described [29]. Briefly, the samples were homogenized in boiling SDS sample buffer (100 mM Tris, 2% SDS, 20% glycerol). The protein concentration was measured using a BCA assay (Pierce, Rockford, IL, USA). 2-Mercaptoethanol and bromophenol blue were added before loading. The extracts were separated using SDS-PAGE (10%) using 20 µg protein per lane and then transferred onto nitrocellulose filters. The filters were blocked in 2.5% milk for 1 h and incubated with rabbit polyclonal CRF-R1 antibody raised against synthetic 17 amino acid peptide from N-Terminus extracellular domain of CRF-R1 (MBL, Wobum, MA, USA; MC-1778) or rabbit polyclonal CRF-R2 antibody raised against synthetic peptide corresponding to the extracellular N-terminal domain of CRF-R2 (Sigma; St Louis, MO, USA, C4241) (1:2.000, in 2.5% milk) overnight at 4 °C. After incubation with the secondary antibody (peroxidase-conjugated goat anti-rabbit, 1:40,000, Jackson ImmunoResearch, West Grove, PA, USA) for 2 h at ambient temperature, reactive protein bands were digitally visualized using ECL solutions (SuperSignal West Pico, Thermo Scientific) in ChemiDoc MP Imager. Experiments were performed in groups of 4 animals.

The Western blot bands specific for CRF-R1 (56 kDa) or CRF-R2 (38 kDa) according to their data sheet were quantified by Java Image processing and analysis software (ImageJ, open-source image software downloaded from the web#) [30, 31].

### Receptor selectivity

The most effective dose of i.t. CRF was administered together with different doses of CRF-R2 antagonist K41498 (0.75, 1.5, 3, 5 nmol) or CRF-R1 antagonist NBI35965 (0, 0.001, 0.002, 0.004 nmol) to determine the receptor selectivity of CRF-mediated antinociceptive effects. Then, the most effective dose of i.t. CRF-R2 antagonist urocortin II (0.78 pmol) was administered alone or together with different doses of corresponding CRF-R2 antagonist K41498 (0.75, 1.5, 3, 5 nmol) to determine the receptor selectivity of CRF2-mediated antinociceptive effects.

To examine whether CRF-elicited anti-nociception is opioid-mediated, the most effective doses of i.t. CRF agonist (0.2 µmol) or CRF-R2 agonist Ucn-2 (0.075 µmol) were administered together with the opioid receptor antagonist naloxone (0.07, 0.14, 0.42 µmol).

### Immunohistochemistry

#### Tissue preparation

Four days after FCA inoculation, rats were deeply anesthetized with isoflurane and transcardially perfused with 100 ml warm saline, followed by 300 ml 4% (w/v) paraformaldehyde in 0.16 M phosphate buffer solution (pH 7.4). After perfusion, spinal cord (L4-5) and brain were removed, postfixed in the same fixatives for 90 min, and then cryoprotected overnight at 4 °C in PBS containing 10% sucrose. The tissues were then embedded in tissue-Tek compound (OCT, Miles Inc. Elkhart, IN, USA) and frozen. Spinal cord and brain were serially cut at 40 µm on cryostat. Every fourth section of spinal cord and brain was collected in PBS (floating sections).

#### Immunofluorescence staining

For single or double immunofluorescence, tissue sections were processed as described previously [18]. Briefly, coronal or parasagittal spinal cord and brain sections were incubated with rabbit polyclonal antibody against CRF-R1 or CRF-R2 alone or in combination with guinea pig polyclonal antibody against CGRP (1:1000, Peninsula Laboratories, Belmont, CA, USA), mouse monoclonal antibody against ENK (1:1000) or guinea pig polyclonal antibody against MOR (1:1000, Chemicon International, MA, USA) as well as with guinea pig polyclonal antibody against CGRP in combination with rabbit anti-rat MOR (dilution of 1:1000) overnight at 4 °C. After incubation with primary antibodies, the tissue sections were washed with PBS and then incubated with Texas Red conjugated goat anti-rabbit antibody (Vector Laboratories) in combination with Alexa Fluor 488 goat anti-guinea pig or anti-mouse antibody (Invitrogen, Germany). Thereafter, sections were washed with PBS, and the nuclei stained bright blue with 4'-6-Diamidino-2-phenylindole (DAPI) (0.1 µg/ml in PBS) (Sigma). Finally, the tissues were washed in PBS, mounted in vectashield (Vector Laboratories) and imaged on a confocal laser scanning microscope, LSM510 (Carl Zeiss, Göttingen, Germany). To demonstrate specificity of staining, the following controls were included as mentioned in detail elsewhere [18, 20, 32, 33]: (1) pre-absorption of diluted antibody against ENK, MOR with a synthetic peptide for ENK (Peninsula laboratories) or MOR (Gramsch Laboratories), respectively; (2) omission of either the primary antisera or the secondary antibodies.

### 2.11 Analysis of data

Data were analyzed using one-way ANOVA followed Dunnett’s post hoc test. For data not normally distributed, Kruskal–Wallis One Way Analysis of Variance on ranks was performed, followed by Dunnett’s or Tukey post hoc test. Dose–response curves were analyzed by one-way ANOVA followed by linear regression. Differences were considered significant if P < 0.05. All tests were performed using Sigma Stat 2.03 (SPSS Science, Chicago, IL. USA) software. Data are expressed as means ± SD or means ± s.e.m.

## Results

### Distinct expression of spinal CRF-R1 and CRF-R2 receptors

**Fig. 1:**
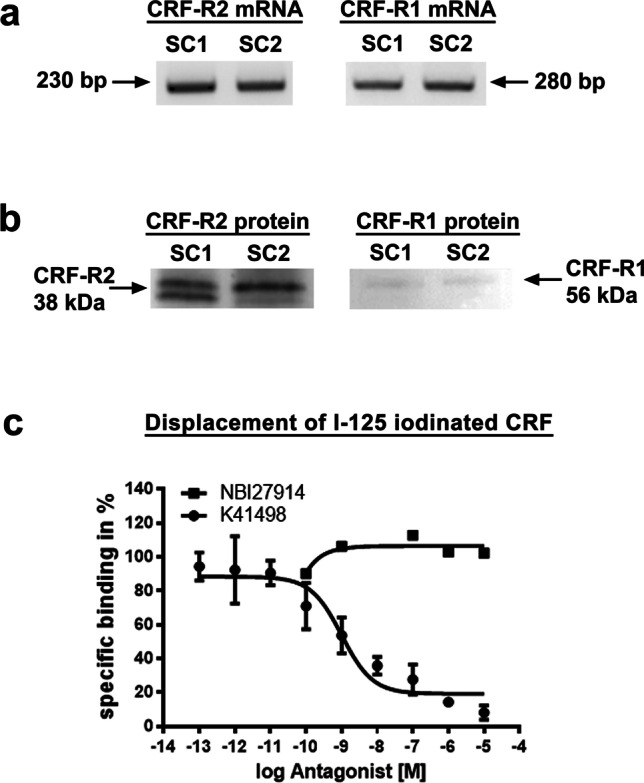
Demonstration of CRF-R1 and CRF-R2 mRNA and protein as well as specific binding sites in the dorsal part of the spinal cord. (**a**) RNA extraction from the dorsal part of the L4-L5 spinal cord, implementation of conventional PCR using specific primer pairs for CRF-R1 and CRF-R2, and subsequent visualization on a 2% agarose gel provided specific PCR products for the expression of CRF-R1 (280 bp) and CRF-R2 (230 bp) mRNA. (**b**) Western blot analysis of rat L4-L5 spinal cord using specific antibodies against CRF-R1 and CRF-R2 revealed protein bands at the expected molecular weight of 56 kDa for CRF-R1 and 38 kDa for CRF-R2. Although the same amount of protein was loaded (20 µg) onto the gel, the protein band of CRF-R2 was much more prominent than that of CRF-R1. (**c**) Displacement of [^125^ J]-CRF- binding by unlabelled CRF-R1- (NBI35965) and CRF-R2- (K41498) selective ligands shows clearly identifiable displacement of [^125^ J]-CRF-binding with increasing concentrations of the CRF-R2-selective antagonist K41498 (IC50 = 500 pmol), however, a lack of displacement by the CRF-R1 selective antagonist NBI35965. The curves are fits to a single-site inhibition equation. Data points (n = 6) represent means ± s.e.m

Using a highly specific primer pair, both CRF-R1 and CRF-R2 mRNA were detectable in the dorsal part of the spinal cord (Fig. 1a). Concurrently, protein precipitation and separation of the dorsal part of the spinal cord (L4–L5) via gel electrophoresis and subsequent western blot revealed prominent CRF-R2 protein bands at the expected molecular weight (38 kDa), whereas CRF-R1 protein bands (56 kDa) were only faintly detectable (Fig. 1b).

Consistent with these findings, displacement experiments of radiolabelled [^125^ J]-CRF-binding by CRF-R1- (NBI35965) and CRF-R2- (K41498) selective antagonists in membranes of the dorsal part of the spinal cord demonstrated clearly identifiable displacement with increasing concentrations of the CRF-R2-selective antagonist K41498 (IC_50_ = 500 pmol) (Fig. 1c), yet a lack of displacement with the CRF-R1 selective antagonist NBI35965 (Fig. 1c).

**Fig. 2:**
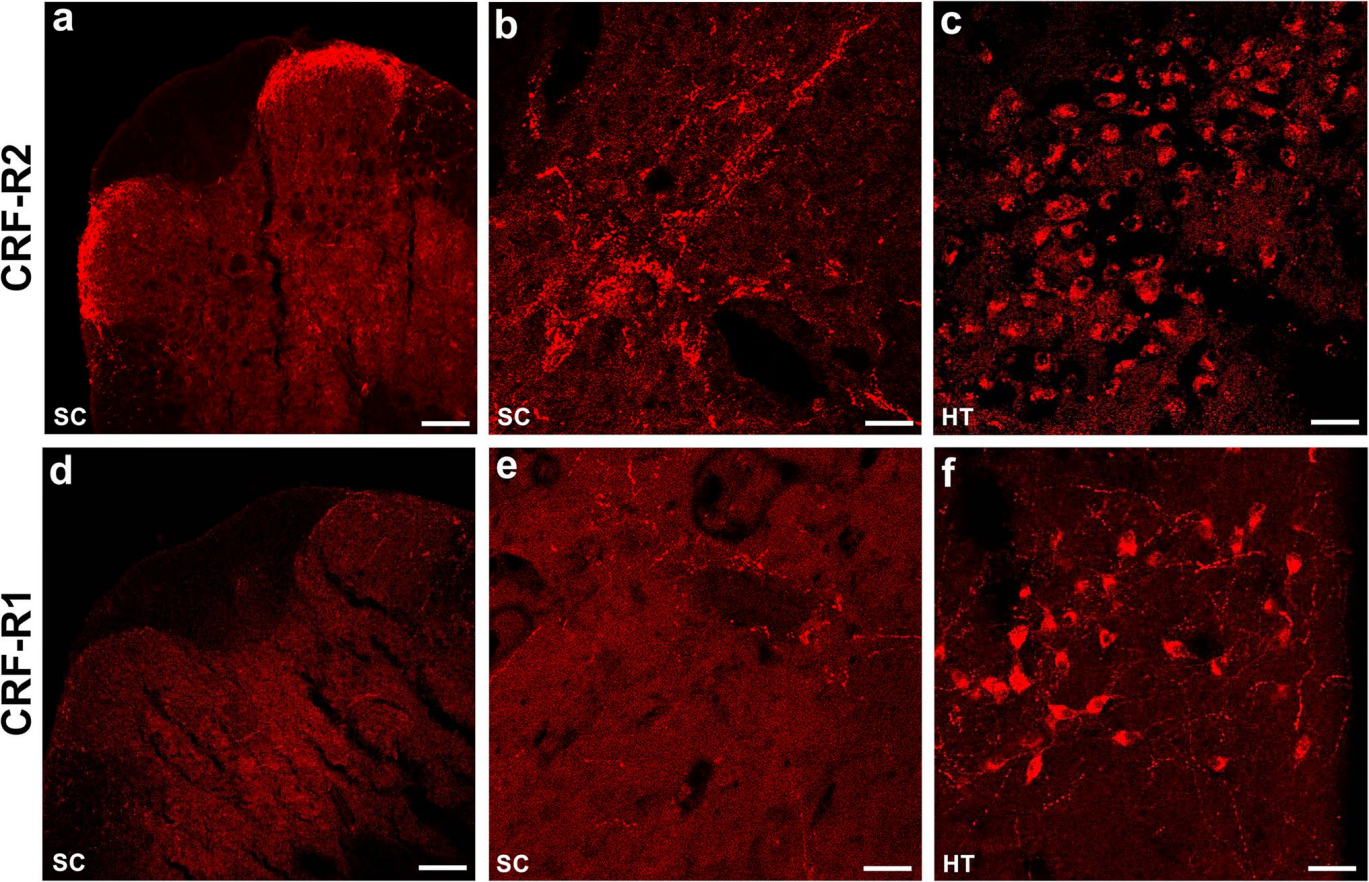
Immunofluorescence staining of CRF-R2 (**a**–**c**) and CRF-R1 (**d**–**f**) in the superficial laminae of the rat spinal dorsal horn as well as the brain. (**a**–**c)** Immunohistochemical staining of the dorsal horn of the spinal cord (**a**, **b**) and of the hypothalamus of the brain (**c**) with the polyclonal rabbit anti- CRF-R2 antibody (*Texas red fluorescence*). Note specific CRF-R2 immunoreactivity in Rexed laminae I and II of the spinal dorsal horn (**a**) and in the immediate vicinity of the central canal (**b**), an area receiving descending projections from supraspinal nuclei. CRF-R2 immunoreactivity was also identified in neurons of the hypothalamus (**c**). (**d**–**f**) Immunohistochemical staining of the dorsal horn of the spinal cord (**d**, **e**) and of the hypothalamus of the brain (**f**) with the polyclonal rabbit anti-CRF-R1 antibody (*Texas red fluorescence*). In contrast to CRF-R2, CRF-R1 immunoreactivity was not identified in the spinal dorsal horn (**d**) and only scarce around the spinal central canal (**e**), whereas CRF-R1 was abundantly shown in neurons of the hypothalamus (**f**). Bar = 40 µm (A, D), Bar = 20 µm (B, C, E, F)

In cross-sectional spinal L4–L5 segments of naïve rats CRF-R2 immunoreactivity was strongly visible, whereas CRF-R1 immunoreactivity was only scarce (Fig. 2). CRF-R2 immunoreactivity was predominantly detectable in Rexed laminae I and II on both sides of the spinal dorsal horn (Fig. 2a) and in the immediate vicinity of the central canal (Fig. 2b), an area receiving descending projections from supraspinal nuclei [34], whereas CRF-R1 immunoreactivity was not detectable in Rexed laminae I and II (Fig. 2d) and was only scarce around the central canal (Fig. 2e). CRF-R1 (Fig. 2f) as well as CRF-R2 (Fig. 2c) immunoreactivity, however, were clearly detectable in brain areas of known CRF-R1 expression such as hypothalamus confirming the specificity of the antibody [35, 36].

### Distinct contribution of CRF-R2 and CRF-R1 receptors to the antinociceptive effects of intrathecal CRF in inflamed hind paws

**Fig. 3:**
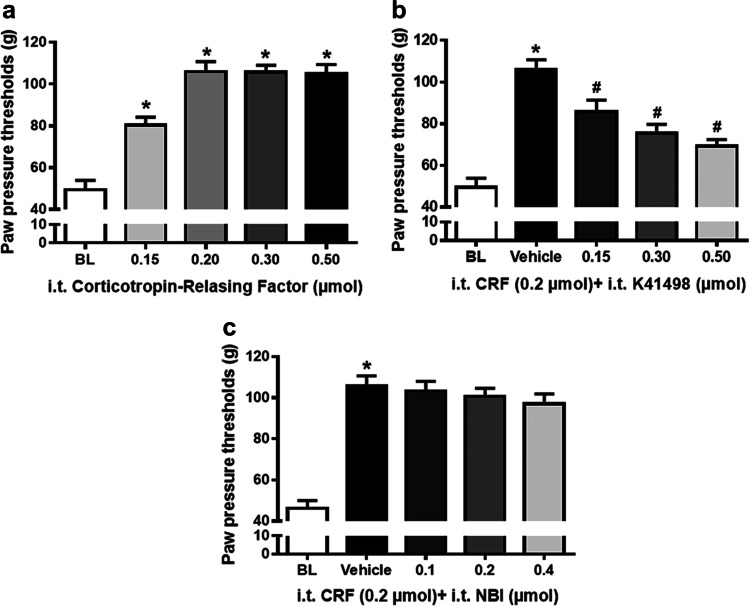
Antagonism of antinociceptive effects of i.t CRF by CRF-R2 (K41498) but not CRF-R1 (NBI35965) antagonist. In Wistar rats with four days FCA-induced inflamed hind paw, effects of intrathecal (i.t.) injections of CRF on nociceptive paw pressure thresholds (PPT) were measured by algesiometer. (**a**) Intrathecal injections of CRF (0.15, 0.2, 0.3, 0.5 µmol) significantly increased PPT in a dose-dependent manner (*P* < 0.001, one-way ANOVA and post-hoc Dunnett’s test, *indicates significant differences from 0, i.e. vehicle treatment). Data points (n = 6) represent means ± SD. (**b**) Dose-dependent antagonism of i.t. CRF’s (0.2 µmol) anti-nociception by co-administered CRF-R2 antagonist K41498 (0.15, 0.3, 0.5 µmol) was significant (*P* < 0.001, one-way ANOVA and post-hoc Dunnett’s test, *indicates significant differences from 0, i.e. vehicle treatment; ^#^indicates significant differences from CRF 0.2 µmol + vehicle). Data points (n = 6) represent means ± SD. (**c**) Intrathecal injection of 0.2 µmol CRF significantly increased PPT compared to vehicle (*P* < 0.001, one-way ANOVA and post-hoc Dunnett’s test, *indicates significant differences from 0, i.e. vehicle treatment); however, increasing doses of the i.t. CRF-R1 antagonist NBI35965 (0.15, 0.2, 0.4 µmol) did not antagonize CRF’s (0.2 µmol) anti-nociception (P = 0.072, one-way ANOVA). Data points (n = 6) represent means ± SD

In Wistar rats with an inflamed right hind paw, i.t. administration of increasing doses of CRF significantly and dose-dependently increased paw pressure thresholds (PPT), (*P* < 0.001, one-way ANOVA and post-hoc Dunnett’s test) (Fig. 3a). This anti-nociceptive effect of i.t. CRF was antagonized with increasing i.t. doses of the CRF-R2 receptor selective antagonist K41498 (*P* < 0.001, one-way ANOVA and post-hoc Dunnett’s test) (Fig. 3b), but not with the CRF-R1 receptor selective antagonist NBI35965 (*P* = 0.072, one-way ANOVA) (Fig. 3c).

**Fig. 4:**
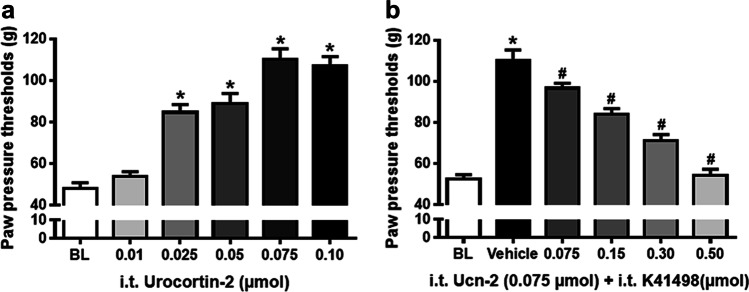
Antinociceptive effects of the i.t. CRF-R2 agonist Ucn-2 and its antagonism by the CRF-R2 selective antagonist (K41498). In Wistar rats with four days FCA-induced inflamed hind paw, effects of intrathecal (i.t.) injections of the CRF-R2 agonist Ucn-2 on nociceptive paw pressure thresholds (PPT) were measured by algesimeter. (**a**) Intrathecal injections of the CRF-R2 agonist Ucn-2 significantly increased PPT in a dose-dependent manner (*P* < 0.001, one-way ANOVA and post-hoc Dunnett’s test, *indicates significant differences from 0, i.e. vehicle treatment). Data points (n = 6) represent means ± SD. (**b**) Intrathecal injection of 0.075 µmol UCN-2 significantly increased PPT compared to vehicle (*P* < 0.001, one-way ANOVA and post-hoc Dunnett’s test, *indicates significant differences from 0, i.e. vehicle treatment); however, dose-dependent antagonism of i.t. Ucn-2 (0.075 µmol) anti-nociception by co-administered CRF-R2 antagonist K41498 (0.075, 0.15, 0.3, 0.5 µmol) was significant (P < 0.001, one-way ANOVA and post-hoc Dunnett’s test, ^#^indicates significant differences from 0, i.e. vehicle treatment). Data points (n = 6) represent means ± SD

To corroborate this finding, animals with an inflamed right hind paw received the CRF-R2 selective agonist Ucn-2 intrathecally which also resulted in dose-dependently increased paw pressure thresholds (*P* < 0.001, one-way ANOVA and post-hoc Dunnett’s test) (Fig. 4a). As expected, this anti-nociceptive effect of i.t. Ucn-2 was antagonized by increasing i.t. doses of the CRF-R2 receptor selective antagonist K41498 (*P* < 0.001, one-way ANOVA and post-hoc Dunnett’s test) confirming CRF-R2 receptor selectivity (Fig. 4b).

**Fig. 5:**
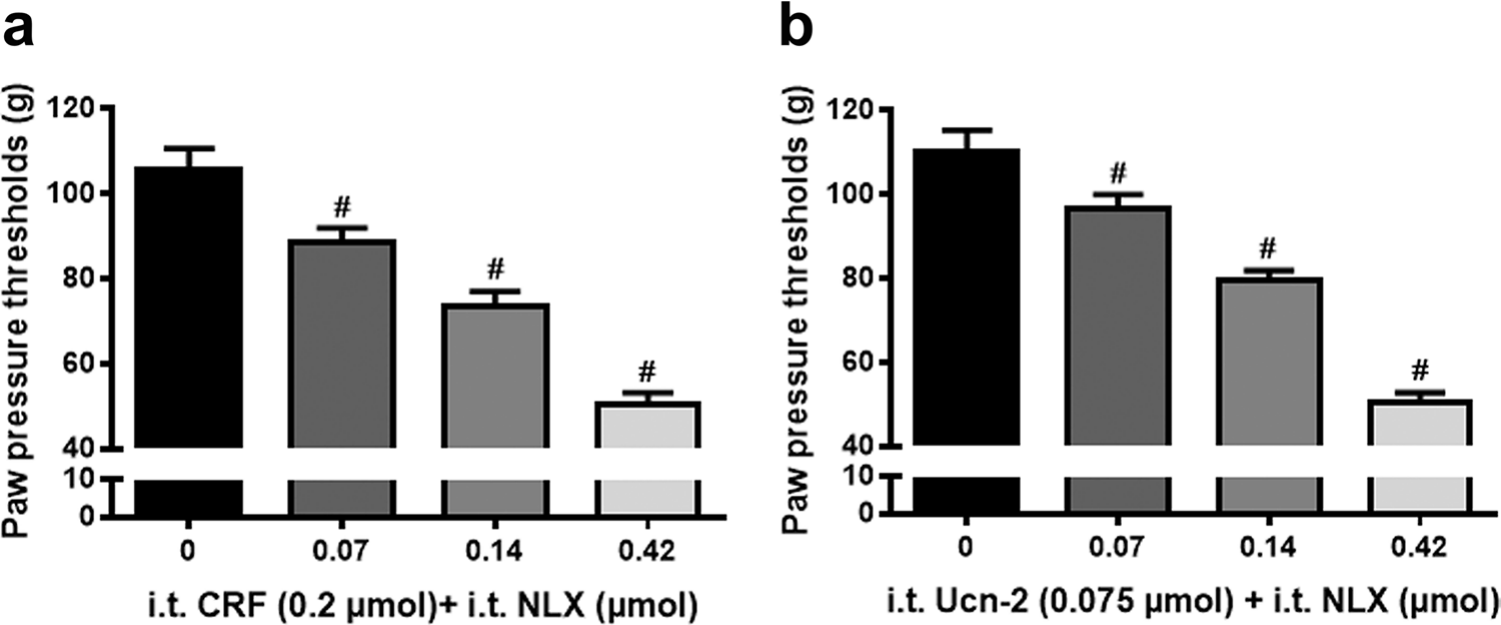
Attenuation of the antinociceptive effects of i.t. CRF and CRF-R2 agonist Ucn-2 by the opioid receptor antagonist naloxone. In Wistar rats with four days FCA-induced inflamed hind paw, effects of i.t. co-administration of the opioid receptor antagonist naloxone with CRF or CRF-R2 agonist Ucn-2 on nociceptive paw pressure thresholds (PPT) were measured by algesimeter. (**a**) Dose-dependent attenuation of i.t. CRF’s (0.2 µmol) anti-nociception by co-administered opioid receptor antagonist naloxone (0.07, 0.14, 0.42 µmol) was significant (P < 0.001, one-way ANOVA and post-hoc Dunnett’s test, ^#^indicates significant differences from CRF (0.2 µmol) + vehicle). Data points (n = 6) represent means ± SD. (**b**) Dose-dependent attenuation of i.t. CRF-R2 agonist Ucn-2 (0.075 µmol) anti-nociception by co-administered opioid receptor antagonist naloxone (0.07, 0.14, 0.42 µmol) was significant (P < 0.001, one-way ANOVA and post-hoc Dunnett’s test, ^#^indicates significant differences from UCN-2 (0.075 µmol) indicates significant differences from 0, i.e. vehicle treatment). Data points (n = 6) represent means ± SD

Intriguingly, i.t. administration of either CRF (Fig. 5a) or Ucn-2 (Fig. 5b) together with the opioid receptor antagonist naloxone dose-dependently diminished the anti-hyperalgesic effect of both substances (*P* < 0.001, one-way ANOVA and post-hoc Dunnett’s test) indicating an opioid receptor-mediated effect.

### Spinal cord areas of CRF receptor and opioid peptide co-expression

**Fig. 6:**
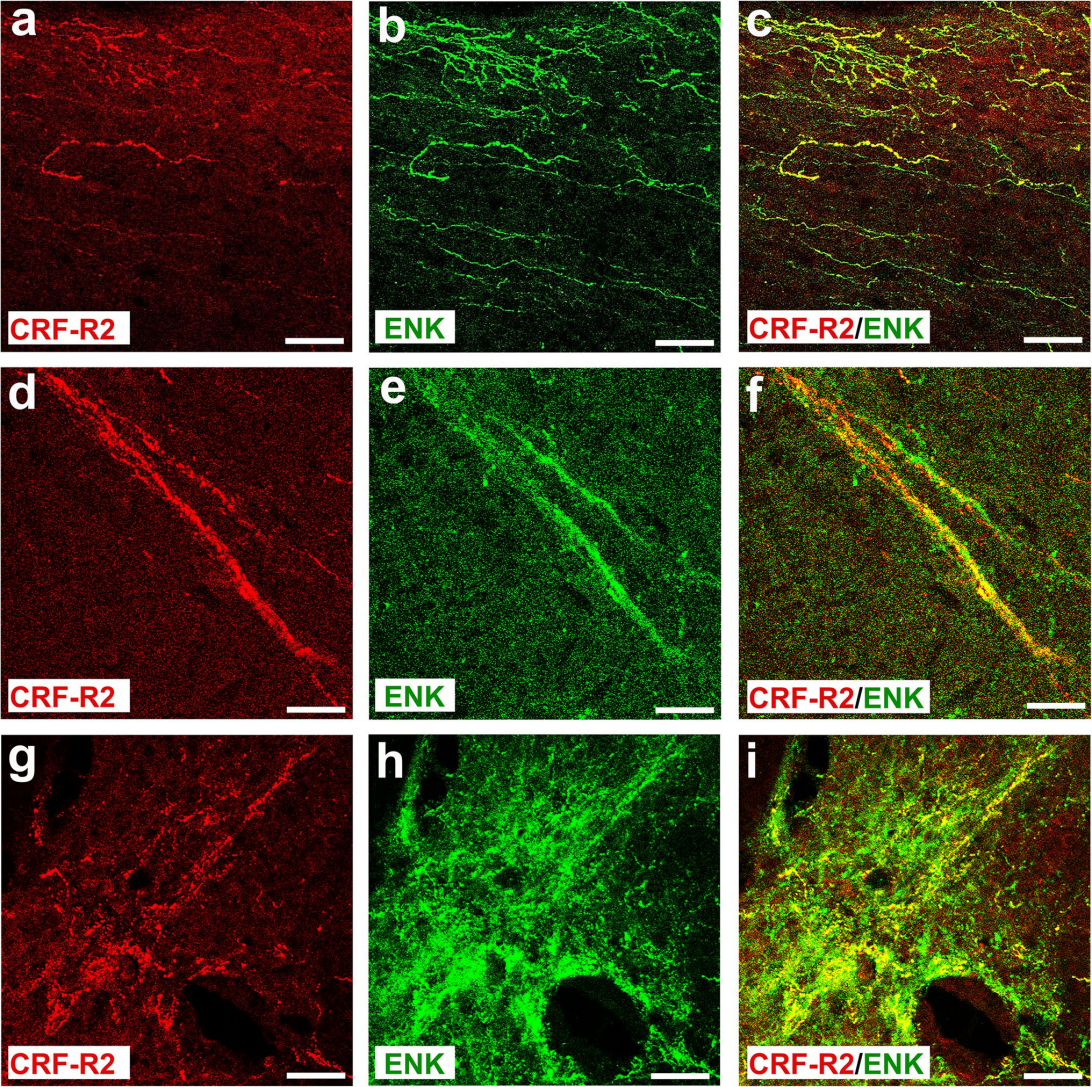
Double immunofluorescence staining of CRF-R2 (**a**, **d**, **g**) and ENK (**b**, **e**, **h**) in the rat L4-L5 spinal cord. (**a**, **b**, **c**) Parasagittal sections of L4-L5 spinal cord of the rat show a network of CRF-R2-immunoreactive fibers overlapping with ENK and extending through the superficial laminae of the dorsal horn of the lumber spinal cord. Some fibers express only ENK. (**d**–**i**) Double immunofluorescence staining of coronal sections of spinal cord of the rat showing that CRF-R2-immunoreactive fibers overlap with ENK. Some fibers express ENK only. Bar = 20 μm

**Fig. 7:**
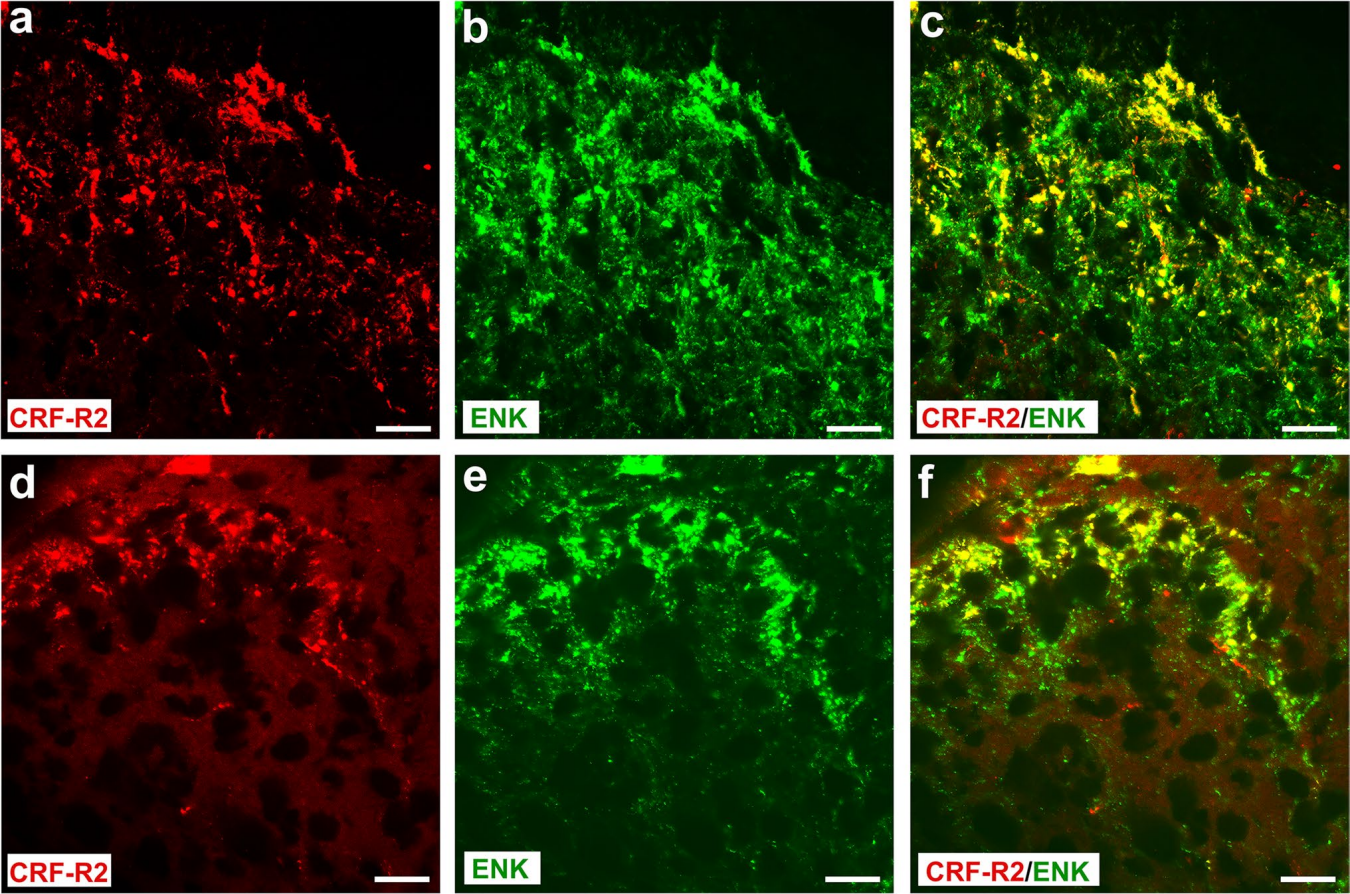
Double immunofluorescence staining of CRF-R2 (**a**, **d**) and ENK (**b**, **e**), in the rat superficial laminae of the L4-L5 spinal dorsal horn. (**a**–**f**) show that most of CRF-R2-immunoreactive fibers express ENK in coronal sections of the L4-L5 spinal dorsal horn of Wistar rats, but few fibers contain CRF-R2 (*Texas red fluorescence*) or ENK (*FITC green fluorescence*) only. Bar = 20 µm

**Fig. 8:**
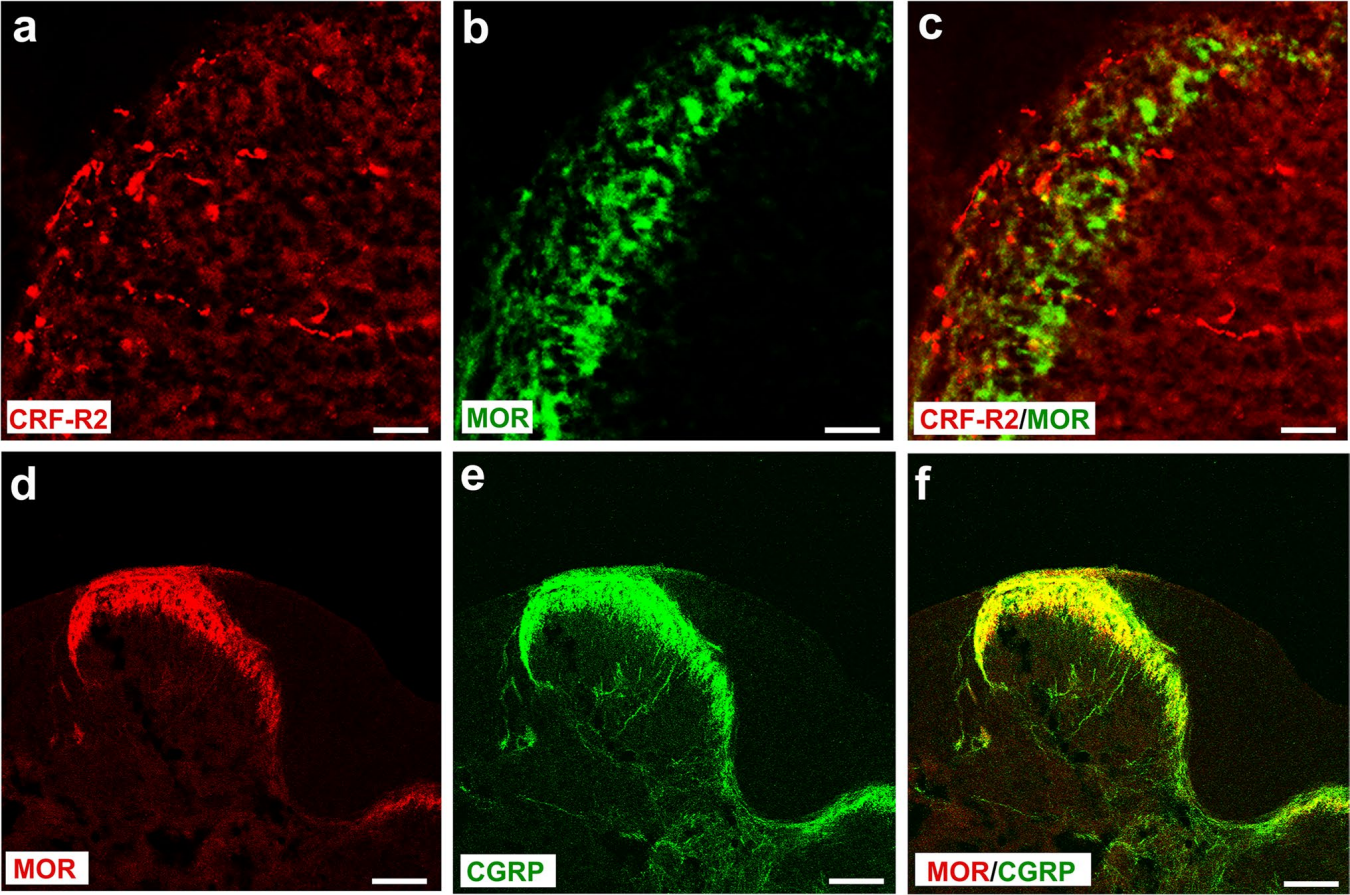
Double immunofluorescence staining of CRF-R2 (**a**) with MOR (**b**) or MOR (**d**) with CGRP (**e**) in the rat superficial laminae of the L4-L5 spinal dorsal horn. (**A**–**C**) show in coronal sections of the L4-L5 spinal dorsal horn that most of CRF-R2-immunorective fibers (*Texas red fluorescence*) (**a**, **c**) do not express MOR (FITC green fluorescence) (**b**, **c**); (**d**–**f**) however, MOR-immunoreactive fibers (*Texas red fluorescence*) (**d**) overlap with CGRP (FITC green fluorescence) (**e**), a neuronal marker of incoming nociceptive nerve fibers. Bar = 20 μm

CRF-R2 immunoreactivity was found in the dorsal horn, the parasympathetic nucleus, and around the central canal of the spinal cord (Fig. 6). Double immunofluorescence confocal microscopy showed that CRF-R2 immunoreactivity within the superficial laminae of the spinal dorsal horn predominantly overlapped with ENK-immunoreactivity (Fig. 7). Since most ENK-IR neurons within the dorsal horn characterize inhibitory interneurons [37–39], our results suggest that CRF-R2 was mainly expressed in inhibitory enkephalinergic interneurons of the dorsal horn of the spinal cord (Fig. 7). These ENK-ir spinal interneurons were found in close proximity of MOR derived from incoming CGRP-ir sensory neurons (Fig. 8).

## Discussion

Extending our previous observations that i.t. CRF significantly diminishes mechanical hyperalgesia in rat inflamed hind paws [18], the present study identified CRF-R2 as the most prominent CRF-receptor subtype within the rat spinal cord which are densely localized in Rexed laminae I and II of the dorsal horn, whereas the CRF-R1 receptors are only scarce. Consistently, spinal receptor binding of I-125 radiolabelled CRF was fully displaced by the CRF-R2- but not by the CRF-R1-selective antagonist. Our behavioral experiments in rats with FCA-induced hind paw inflammation showed that i.t. CRF-induced antinociceptive effects were fully antagonized by the CRF-R2 (K41498) but not by the CRF-R1 (NBI35965) selective antagonist. The same anti-nociceptive effect was obtained by i.t. injection of the CRF-R2 selective agonist Ucn-2 confirming a predominant role of spinal CRF-R2. Interestingly, CRF-R2-mediated antinociceptive effects were reversible by the opioid antagonist naloxone indicating the involvement of endogenous opioid peptides. Indeed, double immunofluorescence confocal microscopy of the spinal cord showed a dense co-localization of CRF-R2 with ENK-containing interneurons in close opposition of corresponding MOR from incoming sensory neurons. Together, these findings suggest that CRF-R2 located on spinal enkephalinergic inhibitory interneurons are the most prominent CRF-receptor subtype involved in endogenous opioid-mediated inflammatory pain inhibition.

Spinal cord isolation of poly-A-tail mRNA and conventional reverse-transcriptase PCR with primers specific for CRF-R2 and CRF-R1 revealed nucleotide bands of the expected sizes confirming the expression of both receptors in the lumbar part of the spinal cord. While Sosanya et al. [40] also demonstrated abundant CRF-R2 mRNA in the spinal cord of Sprague Dawley rats, they were unable to detect CRF-R1 mRNA. CRF-R2 mRNA was also confirmed in the rat spinal cord by reverse-transcriptase-polymerase chain reaction [10], while [41] Korosi et al. reported the presence of both CRF-R1 and CRF-R2 mRNA in the mouse spinal cord using in-situ hybridization. Intriguingly, evidence from studies in the pituitary gland [42] and human synoviocyte cells [41, 43] revealed that CRF receptor expression is under the control of certain transcription factors: while c-Jun/AP-1 transcription factors delivered negative control, HIF-1α and NF-κB delivered positive control of CRH-R1. In our Western blot experiments the respective protein bands for both receptors were detectable at their expected molecular weight, however, the protein band for CRF-R2 was much more prominent, while that for CRF-R1 was only weakly detectable indicating a considerably higher density of spinal CRF-R2 than CRF-R1 receptors. A very low expression of spinal CRF-R1 — as indicated by the western blot results — could be an explanation for the fact why in our radiolabeled ligand binding experiments spinal [^125^ J]-CRF binding sites were fully displaced by the selective CRF-R2 ligand (K41498) but not by the selective CRF-R1 ligand (NBI35965**)**. Our results extend previous autoradiographic CRF receptor studies in the rat spinal cord [44, 45] demonstrating a predominance of CRF-R2 over CRF-R1 binding cites. Consistently, immuno-fluorescence staining of rat spinal cord showed that CRF-R2 was mainly distributed in the superficial laminae of the dorsal horn, whereas CRF-R1 immunoreactivity was only scarcely distributed throughout the spinal cord, confirming previous autoradiographic studies [45]. Taken together, our findings of CRF-R2 receptor protein and membrane binding sites strongly support the notion that CRF-R2 is the dominant CRF receptor subtype in rat spinal cord.

Extending our previous findings [18], the present behavioral experiments revealed that i.t. CRF-induced anti-nociception was dose-dependently attenuated by the selective CRF-R2 (K41498) but not by the CRF-R1 (NBI35965) antagonist. Consistently, i.t. application of the CRF-R2 agonist Ucn-2 dose-dependently and receptor-selectively reduced the inflammation-evoked nociception. Originally, Hargreaves et al. [5] demonstrated for the first time that i.v. CRF elicited an antinociceptive effect in rats similar to that of morphine. In a clinical trial, dental surgery patients having received i.v. CRF 60 min after third molar extraction exhibited significantly less postoperative pain compared to placebo [5]. A central role of CRF in nociceptive behavior was further supported by Lariviere et al. [19, 46], particularly under stressful conditions. In addition, more recent studies supported the role of spinal CRF-R2 in visceral pain [10, 40, 47] or in stress-induced gastric hyperalgesia [48]. Consistently, colorectal distension-induced ERK1/2-phosphorylation in dorsal horn neurons of spinal laminae I and II was attenuated by the CRF-R2 agonist Ucn-2 [10].

In line with our previous study [18], i.t. application of the opioid receptor antagonist naloxone reversed the antinociceptive effects of CRF. Extending these findings, our present behavioral experiments showed that the anti-nociceptive effect of i.t. CRF-R2-selective agonist Ucn-2 was attenuated by naloxone. This suggests that anti-nociception of i.t. CRF or CRF-R2 agonist Ucn-2 seems to be mediated by activation of opioid receptors through endogenous opioid peptides within the spinal cord. In line with our behavioral findings, our double immunofluorescence confocal microscopy analysis showed that most of CRF2-IR nerve fibers overlap with ENK-IR interneurons but not with primary afferent (CGRP-IR) central endings or MOR-ir nociceptive neurons in the superficial dorsal horn of the spinal cord. Consistent with a release of opioid peptides, our results show that the ENK positive inhibitory interneurons are in close proximity of clusters of MOR-IR nociceptive neurons located on incoming CGRP-ir nociceptive neurons [49] and their activation may contribute to presynaptic-inhibition of sensory neuron neurotransmitter release [4, 50, 51] and/or postsynaptic hyperpolarization of excitatory neurons [52]. Therefore, we hypothesized that i.t. CRF administration activates CRF-R2, predominantly expressed in inhibitory interneurons within the dorsal horn of spinal cord, which subsequently may cause endogenous ENK release from interneurons and consequently inhibits inflammatory pain through opioid receptor activation [4, 18]. Indeed, this explanation was supported by the inhibition of CRF-induced anti-nociception within the spinal cord by the i.t. opioid receptor antagonist naloxone.

## Conclusion

In summary, the current study demonstrates that the i.t. application of CRF or the CRF-R2 agonist Ucn-2 elicits potent anti-nociception in an animal model of persistent inflammatory pain. This effect is dose-dependent and attenuated by the CRF-R2 (K41498) but not by the CRF-R1 (NBI35965) selective antagonist, indicating CRF-R2 receptor selectivity. Moreover, i.t. opioid receptor antagonist naloxone dose-dependently reversed either CRF’s or CRF-R2 agonist’s antinociceptive effects at the spinal level of pain transmission. Consistently, we have identified ENK-immunoreactive inhibitory interneurons which co-express CRF-R2 within the dorsal horn of the spinal cord. The present results highlight the need for further studies to investigate the pain modulatory role of CRF-R2 that is mediated by spinal endogenous opioids during inflammatory pain.

## Supplementary Information

Below is the link to the electronic supplementary material.

Supplementary file1 (JPG 3 kb)

## Data Availability

The datasets used and/or analyzed during the current study are available from the corresponding author (Shaaban.mousa@charite.de) on reasonable request. The authors will take responsible for maintaining availability.

## Abbreviations

CRF: Corticotropin releasing factor
CRF-R1: CRF receptors subtypes 1
CRF-R2: CRF receptors subtypes 2
ENK: Enkephalin
FCA: Freund’s complete adjuvant
i.t.: Intrathecal
PPT: Paw pressure thresholds
DRG: Dorsal root ganglia
PCR: Polymerase chain reaction
