# Supplementary figures and images for: Functional and Anatomical Characterization of Corticotropin-Releasing Factor Receptor Subtypes of the Rat Spinal Cord Involved in Somatic Pain Relief
Source: Mol Neurobiol. 2021 Jul 31;58(11):5459–72. doi: 10.1007/s12035-021-02481-z (PMC8599353; doi:10.1007/s12035-021-02481-z)

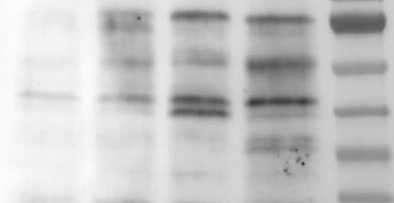

Supplement: Supplementary file 1 — Supplementary file1 (JPG 3 kb) [file 12035_2021_2481_MOESM1_ESM.jpg]
